# Leadless Pacemakers: State of the Art and Selection of the Ideal Candidate

**DOI:** 10.2174/1573403X19666230331094647

**Published:** 2023-07-17

**Authors:** Evan A. Blank, Mikhael F. El-Chami, Nanette K. Wenger

**Affiliations:** 1 Division of Cardiology, Section of Electrophysiology, Emory University School of Medicine, Atlanta, Georgia;; 2 Division of Cardiology, Emory University School of Medicine, Atlanta, Georgia

**Keywords:** Leadless pacemaker, transvenous leads, transvenous pacemaker, cardiac implantable electronic device, safety, patient selection

## Abstract

The field of cardiac pacing has been defined by constant development to provide efficacious, safe, and reliable therapy. Traditional pacing utilizes transvenous leads, which dwell in the venous system and place patients at risk for complications, including pneumothorax, bleeding, infection, vascular obstruction, and valvular compromise.

Leadless pacemakers have been developed to overcome many of the challenges of transvenous pacing while providing safe and effective pacing therapy for an increasing population of patients. The Medtronic Micra transcatheter pacing system was approved by the FDA in April of 2016 and the Abbott Aveir pacemaker was approved in April of 2022. Several additional leadless pacemakers are in various stages of development and testing. There exists limited guidance on the selection of the ideal candidate for leadless pacemakers.

Advantages of leadless pacemakers include decreased infection risk, overcoming limited vascular access, and avoidance of interaction with the tricuspid valve apparatus. Disadvantages of leadless pacemakers include right ventricular-only pacing, unclear lifecycle management, cost, perforation risk, and lack of integration with defibrillator systems. This review aims to provide an overview of the current state of the art of leadless pacemakers, currently approved systems, clinical trials and real-world evidence, considerations for patient selection, and future directions of this promising technology.

## 1 INTRODUCTION

From the inception of cardiac pacing, constant innovation has driven device development to improve the efficacy, reliability, and safety of pacing therapy. The first pacemakers developed in the late 1920s required needles to be percutaneously inserted into a patient’s ventricle. The first transvenous pacemaker for a complete heart block was developed in Sweden in 1958 [1]. Since then, approximately 1.25 million pacemakers have been implanted worldwide each year and their use continues to grow [2].

The widespread utilization of transvenous pacemaker technology has led to the identification of several shortfalls of this pacing approach. Short-term complications of transvenous pacing occur in up to 12% of patients. These complications include bleeding, infection, pneumothorax, lead dislodgement, cardiac perforation, pocket infection, and hematoma. Long-term complications including lead failure, infection, venous obstruction, tricuspid regurgitation, and pocket erosion/infection have been identified in up to 9% of patients [3, 4].

Leadless pacemakers (LP) hold the promise to reduce many short and long-term complications of transvenous pacemaker placement in properly selected patients. In this paper, we discuss the commercially available leadless pacemakers and considerations for patient selection.

## 2 DESCRIPTION OF CURRENTLY APPROVED SYSTEMS

A leadless pacemaker is an entirely self-contained device capable of pacing the heart. At present, two leadless pacemakers are available in the US market.

The Micra transcatheter pacing system (TPS) (Medtronic) was first approved by the FDA in April, 2016 [5]. The Micra is 25.9 mm in length, 0.8cc in volume, and weighs 2 grams (Fig. **1a**). It has 4 nitinol tines that fix it to the myocardium and an accelerometer to allow for rate-responsive pacing. It is delivered with a percutaneous catheter-based approach, typically from the femoral vein. The introducer sheath is 27 French outer and 23 French inner diameter [6].

The initial Micra system was designed as a single-chamber ventricular (VVI) pacemaker. Subsequently, using an enhanced accelerometer-based algorithm downloaded to the device, atrial tracking has been demonstrated to be feasible. With this software upgrade, atrial tracking allows for atrial sensing and ventricular pacing known as VDD pacing. This allows the patient to maintain AV synchrony over a defined range of heart rates (up to a sinus rate of ≅ 105 bpm) and has expanded the potential patient population for the device [7].

The Aveir VR (Abbott) pacemaker was approved by the FDA in April, 2022 [8]. The Aveir VR LP is 38 mm in length, less than 3 grams, and delivered by a sheath with a 25 French inner diameter (Fig. **1b**). It has an active fixation helix, which allows for mapping for R wave sensing, impedance, and initial threshold prior to fixation. It features a docking button and a dedicated system designed for system retrieval [9].

## 3 REVIEW OF THE DATA

The Micra system has been evaluated in several large prospective multicenter studies for efficacy and safety (Table **1**). Micra TPS investigational device exemption (IDE) trial was a global multicenter prospective study to evaluate the efficacy and safety of this device. A total of 725 patients in whom RV pacing was indicated were enrolled, and 99.2% of patients had the device successfully implanted. Device complications in this trial included cardiac perforation at 1.5%, vascular complications at 0.7%, and venous thromboembolism at 0.3%. Subsequent follow-ups of these patients demonstrated freedom from major complications at 96% at 12 months. This freedom from complication rate compared favorably to historical controls with transvenous pacing, with 48% fewer complications. Based on device performance over 24 months, the projected battery life was 12.1 years [6].

The results of the IDE trial were reproduced in the Micra post-approval registry (PAR). In this real-world registry, Micra implantation was attempted in 1826 patients. The implant success rate was 99%. The rate of complications at 6 months was 2.7%, reflecting a 63% reduction in complications as compared to a historical cohort of patients implanted with a transvenous device. The rate of pericardial effusion was 0.77% (0.4% met the definition for major complications). This represented a significant reduction in the rate of perforation as compared to the original Micra IDE study. In Micra PAR, 64% of Micra devices were placed on the septum, while this occurred in 33% of Micra devices in the IDE study. Targeting the septum could have resulted in a lower perforation rate [10].

The Micra Continuous Evidence Development (CED) study is a CMS-mandated claim study, designed to monitor the performance of the LP while under a national coverage determination. In this study, all medicare beneficiaries receiving a Micra implant or a single chamber transvenous study were enrolled and their outcomes were determined using claim data. A total of 5,746 patients implanted with a Micra and 9,662 patients with a transvenous single chamber pacemaker have been enrolled in this study to date. The rate of acute (30 days) adjusted complications was similar between the 2 groups (7.7% *vs.* 7.4%, P = 0.49). Micra was associated with a higher rate of pericardial effusions (0.8% *vs.* 0.4%, P = 0.004) but a lower rate of device-related complications (infection, dislodgment, *etc.*) (1.4% *vs.* 2.5%, P<0.001) as compared to patients implanted with transvenous pacemakers. In addition, patients implanted with leadless pacemakers had a lower rate of complications at 6-month follow-up (HR, 0.77, 95% CI, 0.62-0.96, P = 0.020). This was mainly driven by a reduction in the need for device revision with leadless devices [10].

The 2-year follow-up of the Micra CED study has been recently published [11]. Patients implanted with a leadless device had fewer reintervention as compared to patients implanted with a transvenous VVI system (adjusted hazard ratio (HR) 0.62, 95% confidence interval (CI) 0.45-0.85, P = 0.003) and a lower rate of chronic complications (adjusted HR 0.69, 95% CI, 0.60-0.81, P < 0.0001), highlighting the potential benefits of the leadless system.

Data from the MARVEL 2 study to assess the efficacy of the AV synchronous algorithm demonstrated >70% AV synchrony at rest in 95% of patients with VDD pacing compared to 0% of patients programmed for VVI pacing. The mean percentage of AV synchrony increased from 26.8% during VVI pacing to 89.2% with VDD pacing. Based on these data, the FDA approved the addition of the VDD algorithm.

The first LP to be studied in a clinical trial was the Nanostim (St. Jude Medical) in 2013 [12]. However, the device was removed from the market due to premature battery depletion and reports of spontaneous docking button separation [13].

The LEADLESS II trial - Phase 1 was the main trial that led to Conformité Européenne (CE) Mark approval of Nanostim. In this study, a total of 526 patients were enrolled. The implant success rate was 95.8%. Device-related serious adverse events occurred in 6.5% at 6 months, including pericardial effusion (1.6%), device dislodgment requiring retrieval (1.1%), and groin complications (1.2%) [12].

The Nanostim LP was redesigned and re-named Aveir VR LP(Abbott). The design changes include the use of standard transvenous battery chemistry (lithium carbon-monofluoride) with 12% longer battery life, altered form factor (10% shorter, 1.5F wider), modified docking button to allow for retrieval, and application-specific integrated circuit (ASIC) chip designed to provide an expandable platform (to later support a dual-chamber pacing system once approved).

The LEADLESS-II Phase 2 study enrolled 200 patients with standard ventricular-only pacing indications. Implantation of the Aveir device was successful in 196 patients (98%). Of the successful implants, 83.2% did not require repositioning. The safety end-point analysis was met in 190 of 198 evaluable patients. Three patients had perforation and developed cardiac tamponade (1.5%), 2 of whom required sternotomy and rescue cardiac surgery. Three pre-mature deployments occurred. Of the 196 patients that underwent successful device implants, 188 (95.9%) met the effectiveness criteria. Of the 8 patients that did not meet the effectiveness criteria, 4 failed the capture threshold and 4 failed the R wave amplitude criteria [14].

## 4 ADVANTAGES

There are several potential advantages of leadless pacemakers when compared to transvenous pacing systems (Table **2**).

### 4.1 Decreased Infection Risk

The transvenous pacemaker infection rate has been estimated at 0.77% for an initial implant and 2.08% for revision or replacement [4]. Pacemaker infection increases morbidity and mortality and results in increased healthcare costs [15]. Leadless pacemakers have the potential to reduce the risk of device-related infections. By virtue of their design, LP eliminates the need for a subcutaneous pocket, hence reducing the major source of device-related infection. LP also has a very small surface area as compared to a transvenous lead, which implies a lower chance of bacterial seeding onto the device. Finally, LP has a high incidence of complete endothelialization as opposed to transvenous leads (which are usually partially endothelialized). This will also reduce the chance of bacterial seeding [16].

No Micra or Nanostim/Aveir infections have been reported in any of the clinical trials. To date, there have been more than 100,000 Micra implants with only 2 case reports of the removal of an infected Micra [17, 18]. Based on the low infection risk demonstrated in leadless pacemakers, case series have documented Micra placement at the time of infected transvenous device extraction. Therefore, LP might be preferred in patients at high risk for infection, such as patients with prior infections, patients with diabetes, or immunocompromised patients [16].

### 4.2 Limited Vascular Access

Upper extremity access for transvenous pacemakers may be limited in patients with multiple prior transvenous devices or subclavian vein occlusion. Patients with end-stage renal disease may also have limited upper extremity access due to vascular catheters. The ability to place an LP from the femoral veins can be utilized to overcome limited vascular access. For this reason, leadless pacemaker implantation is given an IIa recommendation in the 2021 ESC pacing guidelines when no upper extremity venous access exists [19].

There are case reports of LP being implanted through iliac stents [20] as well as through inferior vena cava filters. LPs have also been implanted through the internal jugular veins in patients with limited femoral access [21].

### 4.3 Tricuspid Valve Sparing

**Fig. (2):**
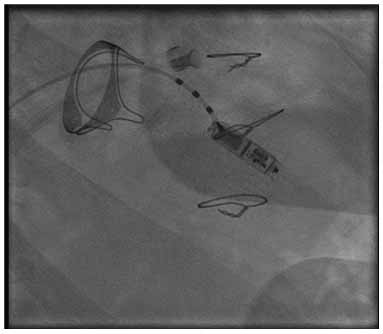
A Micra AV pacemaker is seen inserted through a bioprosthetic tricuspid valve for post-operative heart block.

Leadless pacemakers may also be preferred in patients with tricuspid valve issues, such as severe tricuspid valve regurgitation or tricuspid valve replacement. A leadless pacemaker prevents the possibility of tricuspid valve dysfunction that may occur when a ventricular lead is present across the tricuspid valve. Case series report the use of leadless pacemakers across bioprosthetic tricuspid valves [22] (Fig. **2**).

## 5 LIMITATIONS

There are several limitations and problems that may be encountered with LPs (Table **2**).

### 5.1 Right Ventricular Only Pacing

A limitation of the current LP design is that it provides only the right ventricular pacing. This makes the device poorly suited for patients who require atrial pacing or cardiac resynchronization. Additionally, recent data suggest that conduction system pacing either *via* His bundle or left bundle branch area pacing may be preferred to typical apical or septal pacing which is achieved with LP [23].

The problem of lack of AV synchrony has begun to be addressed with the advent of an AV synchronous algorithm. The algorithm in its present form has important limitations and does not provide perfect AV synchrony. Micra AV loses its ability to provide AV synchrony at faster heart rates.

The Aveir system is designed to be an expandable platform to allow integration with a leadless atrial pacemaker. A leadless atrial pacemaker would allow for both atrial sensing for better AV synchrony, as well as atrial pacing. A leadless atrial pacemaker would expand the indication for LPs to include sinus node dysfunction. The Aveir DR i2i pivotal clinical study is currently enrolling patients to assess the clinical safety and efficacy of a dual chamber LP system [24].

Leadless cardiac resynchronization therapy (CRT) has been achieved with the use of the WiSE-CRT system (EBR Systems) in conjunction with a Micra LP. The WiSE-CRT system is a wireless left ventricular endocardial leadless pacing system with a passive electrode implanted in the LV endocardial wall, which converts ultrasound energy delivered by a subcutaneous system into an electrical impulse. The WiSE-CRT system is currently approved in Europe and undergoing investigation in the United States [25]. A case series of eight patients with an indication for CRT pacing achieved with a Micra LP and WiSE-CRT demonstrated that the WiSE-CRT system was able to synchronize to the Micra RV pacing impulse with an acute QRS reduction achieved in all eight patients (204.38±30.26 *vs.* 137.5±24.75 mS, P=0.0^12^] [26].

### 5.2 Unclear Lifecycle Management

Given the relatively new development of LPs, the management of patients implanted with an LP that is at end of battery life or at the time of upgrade (also referred to as life-cycle management) is not yet clear [27]. It is considered that most patients can be managed by abandoning the old LP and adding a new device (which could be a new LP or CRT or dual chamber system). The number of leadless pacemakers that can be implanted in a human heart over time is unknown. Studies on cadaveric hearts suggest that at least 3 Micra LPs can be implanted in an average size right ventricle [28]. Therefore, for the average patient requiring pacing, it is unlikely that these devices will need to be extracted/removed.

The Nanostim was designed to be extracted easily and the company provides tools whose purpose is to facilitate extraction. In the largest experience of Nanostim extraction, the procedure was successful in 66/73 patients (90.4%) with an overall good safety profile. Two cases of tricuspid valve damage as a result of the extraction were reported [29].

The Micra system can be retrieved/extracted successfully using “off the shelf” tools (snare and a steerable sheath). The current system does not have pre-made tools designed for extraction. Some autopsy reports have described complete encapsulation of the device, which would complicate percutaneous removal [30]. However, there are reports of percutaneous device extraction for up to 4 years [31]. In the largest report of Micra retrieval, 40 Micra were reportedly successfully retrieved. Data on 29 of those were available. No major complications were noted during the retrieval [32]. Progress in battery technology and possibly the ability to recharge these batteries without the need for device replacement might lead to more widespread use of LP [33].

### 5.3 Cost

Data on the cost of LPs to the health care system and cost-benefit analysis of their use remains limited. At present, the cost of a leadless pacemaker is estimated at $10,000 and a transvenous pacemaker at $2500. While the decrease in device complications may partially offset this device cost, the significantly higher cost of a leadless pacemaker may prove a barrier to widespread adoption.

An analysis of the cost and cost-benefit of the use of Micra LP in the Norwegian health system has been undertaken by the Norwegian Institute of Public Health. The analysis calculated an incremental cost-effectiveness ratio (ICER) for the sub-group of patients at high risk for infections (approx. 10-30% of patients requiring pacemakers in the Norwegian system) was NOK 1,077,363 (approx. 105,700 USD). The Micra LP was deemed to not be cost-effective as it exceeded the nation’s prespecified cutoff of NOK 500,000/QALY [34].

### 5.4 Perforation Risk

While the rate of cardiac perforation for LP has been found to be less than 1% (0.77% in the Micra PAR and 0.8% in Micra CED), this rate appears to be higher than with transvenous pacemakers (0.4% in the control group of Micra CED study). In addition, the perforation that occurs during LP implant might be more severe as suggested by a recent analysis of the MAUDE database [35]. Moreover, changes to the delivery system must be made to enhance the safety of this procedure.

### 5.5 Lack of Integration with Defibrillator Systems

Currently approved leadless pacemakers are not designed to be integrated with Implantable Cardiac Defibrillator (ICD) systems. Transvenous ICDs provide backup pacing, obviating the need for an LP. The Emblem Subcutaneous-ICD (S-ICD) (Boston Scientific) enables defibrillation therapy without transvenous hardware. The S-ICD provides similar advantages as leadless pacemakers when compared to transvenous ICD systems. S-ICDs have demonstrated a lower risk of lead-related complications compared to transvenous ICDs [36].

However, the S-ICD system is unable to provide pacing given the subcutaneous location of the ICD lead (except for short post-shock pacing). This precludes the use of S-ICDs for patients with bradycardia indications for pacing. Additionally, the S-ICD system lacks the ability to provide Anti-Tachycardia Pacing (ATP), which has been demonstrated to reduce the need for ICD shocks in the setting of sustained ventricular arrhythmia [37].

The successful implantation of a Micra and S-ICD system has been described in patients with indications for a pacemaker and ICD with limited vascular access [38]. However, the systems are not approved or designed to be co-implanted. Since the systems do not communicate, ATP cannot be delivered. Additionally, there is a theoretical concern that the paced QRS may cause an increase in double counting or T wave oversensing, resulting in inappropriate S-ICD tachyarrhythmia detections and therapy [39].

The MODULAR ATP study is currently investigating the integration of the Empower Modular Pacing System (Boston Scientific) with the EMBLEM Subcutaneous ICD. The EMPOWER MPS is designed to provide both right ventricular bradycardia pacing support as well as ATP triggered by the S-ICD system. The system is backward compatible allowing for existing S-ICDs to be updated with firmware to allow communication with the EMPOWER LP. The communication between a device in the current iteration is one-way. The S-ICD detects tachyarrhythmias *via* the subcutaneous lead and triggers ATP therapy *via* the LP. The S-ICD then continuously monitors for the result of the ATP therapy and delivers a shock if ATP does not terminate the tachycardia [40]. Detections of arrhythmias and response to therapy could potentially be improved in future iterations if the device is designed to communicate in a two-way fashion.

### 5.6 Lack of Randomized Clinical Trials

The benefits of LPs need to be confirmed in randomized clinical trials (RCT). While data from longitudinal registries are helpful, the pros and cons of any therapy are best tested in an RCT.

## 6 PATIENT SELECTION

### 6.1 Leadless Pacemaker as a First Choice

Given the advantages of a leadless pacemaker noted above, there are subsets of patients requiring pacing for whom a leadless pacemaker may be the best choice (Table **3**). Patients with difficult vascular access, such as bilaterally occluded subclavian venous systems, can benefit from the ability to place the system *via* the femoral venous system [41].

Patients who are at high infection risk, such as those with recent bacteremia, recurrent infections, and intravenous drug users may benefit from the very low reported infection rate. Patients on dialysis or who are close to dialysis may benefit by preserving upper extremity vasculature and decreasing infection risk.

Individuals with bioprosthetic tricuspid valves may potentially avoid damage to the valve caused by a pacing lead crossing a prosthetic valve.

### 6.2 Leadless Pacemaker as a Reasonable Choice

A leadless pacemaker can be considered a reasonable choice for any patient who requires only ventricular pacing (Table **3**). This includes patients with atrial fibrillation with a pacing indication, as AV synchrony is not a concern. Additionally, patients with sinus node dysfunction with limited ventricular pacing needs or tachycardia-bradycardia syndrome with post-conversion pauses are reasonable candidates for ventricular-only pacing. A one-stage treatment for AF with poorly controlled rates with an LP placement and atrioventricular node ablation through the femoral access site has been feasible and safe [42].

With the advent of the VDD algorithm, elderly patients with complete heart block are reasonable candidates for LPs as the algorithm can maintain a reasonable percentage of AV synchrony at slower atrial rates.

Leadless pacemakers have also been used as a “bridge” pacemaker in patients undergoing transvenous pacemaker extraction for infection [43, 44].

### 6.3 When to Avoid a Leadless Pacemaker

With the enumerated limitations of current leadless pacemaker technology, there are patients for whom a leadless pacemaker should be avoided at the current time (Table **3**). Patients with an indication for atrial pacing, such as sick sinus syndrome, should not be considered, as leadless atrial pacing is currently not available. The use of ventricular-only pacing in these patients would lead to a decrease in AV synchrony and the subsequent RV-only pacing would put this group at an unneeded risk for pacemaker syndrome when the alternative of atrial pacing is available.

A transvenous system is presently required for patients in whom Cardiac Resynchronization Therapy is indicated. While an entirely leadless CRT has been described above, this technology is still in its infancy without long-term or large patient follow-up. Additionally, patients with an indication for a defibrillator in addition to pacing require a transvenous system at this time.

Young patients with long-term pacing needs may not be considered ideal candidates for leadless pacemakers at present time due to the lack of long-term data on multiple device implants and extractions given a current battery life of about 12 years. However, some experts advocate for LP for younger patients in whom TV leads are prone to fracture and in whom the need for multiple transvenous lead extractions may be expected over a patient’s lifetime.

## 7 FUTURE DIRECTION AND CONCLUSION

The first generation of leadless pacemakers has currently been utilized worldwide with several thousand implanted in clinical trials and more than 100,000 in clinical practice to date. Real-world experience with implantation and follow-up of the Micra system has thus far demonstrated a desirable safety profile in the short and intermediate term. Studies are currently underway to assess the long-term safety and efficacy of these devices.

Ongoing innovation is expected to address the above-mentioned limitations of single ventricle leadless pacing encountered at this time. Future directions of leadless pacing may include dual chamber leadless pacemakers, cardiac resynchronization therapy, and integration with subcutaneous defibrillators to deliver anti-tachycardia pacing. The development of leadless pacemakers by additional manufacturers can be expected to decrease costs as competition increases.

## Figures and Tables

**Fig. (1a):**
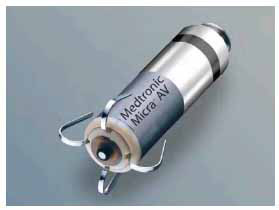
Micra transcatheter pacing system.

**Fig. (1b):**
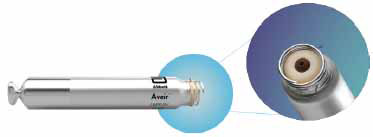
Aveir VR leadless pacemaker.

**Table 1:** Comparison of leadless pacemaker studies.

-	Leadless II- Phase 1 [14]	Leadless II-Phase 2 [15]	Micra IDE [6]	Micra PAR [10]	Micra CED [44]
**N**	526	200	725	1826	5746
**Implant Success (%)**	95.8	98	99.2	99.1	N/A
**Perforation (%)**	1.6	1.5	1.5	0.77	0.8
**Dislodgment (%)**	1.1	1	0	0.05	N/A
**Groin Complication (%)**	1.2	0.5	0.7	0.61	N/A
**Infection**	0	0	0	0	N/A

**Table 2:** Characteristics of leadless and transvenous pacemakers.

**-**	**Leadless ** **Pacemaker**	**Transvenous Pacemaker**
Leads within heart	No	Yes
Implant complications	Low	Significant
Infections	Low risk	High risk
Removable	Less experience	More experience
Atrial pacing*	No	Yes
Cardiac resynchronization*	No	Capable
Cost	High	Low

**Note:** *Might be present with the next generations of devices.

**Table 3:** Patient selection for leadless pacemakers.

**First Choice**	**Reasonable**	**Avoid**
Occluded upper extremity vascular access	Right ventricular only pacing	Atrial pacing needed
End stage renal disease	Bridging pacemaker during lead extraction	Cardiac resynchronization therapy indicated
Recent bacteremia	As part of ablate and pace strategy	Defibrillator indicated
Intravenous drug use	-	Young patients
Bioprosthetic tricuspid valve	-	-
